# C3a Enhances the Formation of Intestinal Organoids through C3aR1

**DOI:** 10.3389/fimmu.2017.01046

**Published:** 2017-09-04

**Authors:** Naoya Matsumoto, Abhigyan Satyam, Mayya Geha, Peter H. Lapchak, Jurandir J. Dalle Lucca, Maria G. Tsokos, George C. Tsokos

**Affiliations:** ^1^Department of Medicine, Beth Israel Deaconess Medical Center, Harvard Medical School, Boston, MA, United States; ^2^Department of Pediatrics, Massachusetts General Hospital, Harvard Medical School, Boston, MA, United States; ^3^Translational Medical Division, Department of Chemical and Biological Technologies, Defense Threat Reduction Agency, Fort Belvoir, VA, United States

**Keywords:** complement 3, intestinal organoid, intestinal stem cell, regeneration, ischemia/reperfusion

## Abstract

C3a is important in the regulation of the immune response as well as in the development of organ inflammation and injury. Furthermore, C3a contributes to liver regeneration but its role in intestinal stem cell function has not been studied. We hypothesized that C3a is important for intestinal repair and regeneration. Intestinal organoid formation, a measure of stem cell capacity, was significantly limited in C3-deficient and C3a receptor (C3aR) 1-deficient mice while C3a promoted the growth of organoids from normal mice by supporting Wnt-signaling but not from C3aR1-deficient mice. Similarly, the presence of C3a in media enhanced the expression of the intestinal stem cell marker leucine-rich repeat G-protein-coupled receptor 5 (Lgr5) and of the cell proliferation marker Ki67 in organoids formed from C3-deficient but not from C3aR1-deficient mice. Using *Lgr5.egfp* mice we showed significant expression of C3 in Lgr5^+^ intestinal stem cells whereas C3aR1 was expressed on the surface of various intestinal cells. C3 and C3aR1 expression was induced in intestinal crypts in response to ischemia/reperfusion injury. Finally, C3aR1-deficient mice displayed ischemia/reperfusion injury comparable to control mice. These data suggest that C3a through interaction with C3aR1 enhances stem cell expansion and organoid formation and as such may have a role in intestinal regeneration.

## Introduction

Intestinal stem cells, also known as crypt base columnar cells, have been recognized as responsible for intestinal regeneration a vital process for the homeostatic self-renewal and the response to catastrophic exogenous insults, including irradiation, exposure to food toxins and ischemia/reperfusion (1). The leucine-rich repeat G-protein-coupled receptor 5 (Lgr5) has been identified as a marker of intestinal stem cells through mouse engineering and cell lineage tracing studies (2). Lgr5 is a target of the Wnt signaling pathway and Wnt agonists, like R-spondin 1, expand Lgr5^+^ cells and accelerate intestinal recovery after irradiation (3). IL-22 supports intestinal regeneration by enhancing the phosphorylation of STAT3 (4), whereas it has been proposed that additional inflammatory signals may be involved in intestinal regeneration.

It has been shown that C3a and C5a are critical in liver tissue regeneration after exposure to carbon tetrachloride or partial hepatectomy by promoting hepatocyte proliferation (5, 6) and in retina tissue regeneration (7). C3a promotes neurogenesis in mice subjected to ischemic brain injury (8) and C3 deficiency decreased the number of cardiac stem/progenitor cells in the infarct zone after coronary artery ligation (9). Furthermore, C3a recruits mesenchymal stem cells to injured tissues through C3a receptor (C3aR) and protects them from oxidative damage (10).

Along these lines, we considered that C3a that is an element of the inflammatory response, contributes to the function of intestinal stem cells and the formation of intestinal organoids by binding to the C3aR1. Here, after we noticed that intestinal organoid formation is limited when crypt cells from C3- and C3aR1-deficient mice were used, we found that C3a is important in promoting intestinal stem cell growth and organoid formation only in mice which express C3aR1. Our data support the concept that C3a is involved in intestinal regeneration by a C3aR1-mediated mechanism and through the expansion of intestinal stem cells.

## Materials and Methods

### Mice

C57BL/6J (B6) mice, C3-deficient mice (B6.129S4-*C3^tm1Crr^*/J), C3aR1-deficient mice (C.129S4-*C3ar1^tm1Cge^*/J), BALB/cJ mice (BALB/c), and *Lgr5.egfp.IRES-CreERT2* knock-in mice (B6.129P2-*Lgr5^*tm1(cre/ERT2)Cle*^*/J) were purchased from Jackson Laboratory. C3-deficient mice were crossed with B6 mice and the offspring were crossed with *Lgr5.egfp* mouse to generate *Lgr5.egfp.IRES-CreERT2* knock-in C3-deficient mice. Eight- to twelve-week-old male mice were used for all experiments. All mice were maintained in the pathogen-free, AAALAC (Association for Assessment and Accreditation of Laboratory Animal Care International) accredited animal facility at Beth Israel Deaconess Medical Center. All experimental protocols were performed in accordance with the National Institute of Health guidelines for the use of experimental animals and were approved by the Institutional Animal Care and Use Committee.

### Isolation of Intestinal Crypts and Intestinal Organoid Culture

Crypts were isolated from the whole small intestine (duodenum, jejunum, and ileum) of 8- to 10-week-old mice and an intestinal organoid culture was initiated as described previously (11), with modifications. After flushing out the feces with ice-cold PBS, the small intestine was opened longitudinally and washed with PBS. The villi were scraped off with a cover slip, and the intestine was divided into pieces. After repeated PBS washings, the intestinal pieces were incubated in 2 mM EDTA-PBS solution and gently rocked at 4°C for 30 min. Subsequently, the EDTA solution was removed and the tissue fragments were vigorously suspended in PBS and filtered through a 70 µm cell strainer. This step was repeated until the fraction was confirmed rich in pure crypts under a microscope. Then, the crypt-rich fractions were centrifuged at 300 *g* for 5 min at 4°C. The pellets were suspended in culture medium described below, centrifuged at 200 *g* for 2 min at 4°C and re-suspended in culture medium. The number of crypts was estimated and crypts were mixed with Matrigel (Corning) at the concentration of 10 crypts/μL. The mixture was transferred into a 24 well plate (50 µL/well). After polymerization of the Matrigel, 500 µL of the culture medium was overlaid on the gel. The standard culture medium was composed of 2 mM Glutamax (ThermoFisher Scientific), 10 mM HEPES, 100 U/mL penicillin, 100 µg/mL streptomycin, 1 mM *N*-acetylcysteine (Sigma-Aldrich), 1× N2 (ThermoFisher Scientific), 1× B27 (ThermoFisher Scientific), 50 ng/mL EGF (ThermoFisher Scientific), 100 ng/mL Noggin (PeproTech), and 1 µg/mL R-spondin 1 (PeproTech) in Advanced DMEM/F12 (ThermoFisher Scientific). Additional reagents necessary to evaluate their effect on the organoid formation were added as follows: C3a protein (2 or 10 µg/mL) (Complement Technology) (12), anti-human C3a/C3 antibodies (13), or the corresponding control mouse IgGs (5 µg/mL) (both Biolegend) in the presence or absence of C3a (2 µg/mL) and Wnt inhibitor IWP-2 (0.2, 0.1, or 0.05 µM) (Stemgent) in the presence or absence of C3a (2 µg/mL). The cultures were maintained in an incubator (37°C, 5% CO_2_). The number of organoids with multiple buds was counted per well 7 days after the initiation of the culture. The fold change was calculated by comparing the number of organoids formed in each experimental versus the corresponding control condition. For quantitative polymerase chain reaction (qPCR) and immunohistochemical analysis, organoids were isolated from the gel 7 days after the initiation of the culture with Cell Recovery Solution (Corning).

### Ischemia/Reperfusion Experiments

Ten- to twelve-week-old B6, C3-deficient mice (C3 KO), BALB/c mice, and C3aR1-deficient mice (C3aR1 KO) were subjected to 30 min ischemia by clamping the superior mesenteric artery followed by 120 min reperfusion. Sham-operated mice underwent the same procedure but without clamping of the artery (14). The intestine was dissected at each time point to isolate the crypts after euthanasia by carbon dioxide.

### Histology and Tissue Injury Scoring

The harvested intestinal tissues were rinsed with PBS and fixed overnight in a 10% phosphate-buffered formalin solution. Formalin-fixed whole small intestine was then embedded in paraffin, sectioned, and stained with hematoxylin and eosin (H&E). Each intestinal tissue was graded blindly by two observers using a published scoring system (15). Briefly, normal villus was assigned a score of 0; development of Gruenhagen’s space only at the apex of the villus usually with capillary congestion, received a score of 1; extension of Gruenhagen’s space with moderate lifting of the epithelial layer from lamina propria was scored as 2; visible breakage of epithelium, but only at the tips of villi, was scored as 3; extensive epithelial damage leading to denuded villi with exposure of intact lamina propria received a score of 4; and extensive mucosal ulceration, as well as necrosis, including digestion and disintegration of lamina propria, was scored as 5. All histologic analyses were performed in a blinded fashion. The images of the sections were captured using a Nikon eclipse 80i microscope and analyzed using Nikon NIS-Elements software (Nikon).

### Real-time PCR (RT PCR)

Total RNA was extracted from organoids or isolated intestinal crypts with the use of RNeasy Plus Mini kit (Qiagen) or TRlzol reagent (ThermoFisher Scientific), respectively. Reverse transcription was performed using RNA to cDNA EcoDry Premix kit (Takara). The resulting cDNA was amplified by TaqMan probe-based PCR (ThermoFisher Scientific) with a LightCycler 480 instrument (Roche). The abundance of each target mRNA was normalized by that of glyceraldehyde 3-phosphate dehydrogenase (GAPDH) mRNA. Taqman primer-probes used were as follows: Ki67 (Assay ID: Mm99999915_g1), Lgr5 (Mm00438890_m1), Lysozyme1 (Mm00657323_m1), Ascl2 (Mm01268891_g1), C3 (Mm01232779_m1), C3aR1 (Mm02620006_s1), and GAPDH (Mm99999915_g1).

### Immunohistochemisty

Organoids or small intestine tissues were immersed in a solution of 4% paraformaldehyde in PBS and cryoprotected in a series of sucrose solutions (15, 20, and 25% sucrose in PBS) at 4°C for 3 days. Subsequently, the specimens were frozen in optimal cutting temperature (OCT) compound (Sakura Finetechnical Co. Ltd.) and sliced into 7-µm sections using a cryostat. For chromogenic detection, endogenous peroxidase activity was quenched with 3% H_2_O_2_ and antigen retrieval was performed in a Tris-EDTA buffer (pH 9.0) with steam heat for 20 min. The sections were blocked for 60 min at room temperature with 10% BSA/PBS containing the serum from the host species of the corresponding secondary antibodies, and incubated overnight at 4°C with a rabbit anti-Ki67 antibody (1:200; Abcam), rabbit anti-Lysozyme antibody (1:500; DAKO), rabbit anti-C3 antibody (1:200; Bioss), goat anti-C3aR1 antibody (1:100; Santa Cruz), and/or chicken anti-GFP antibody (1:1,000; Abcam). The secondary antibodies (1:1,000) were applied at room temperature for 60 min and consisted of horseradish peroxidase (HRP)-conjugated donkey anti-rabbit IgG antibody (Jackson Immuno Research Labs) followed by visualization with NovaRED (Vector Laboratories) for chromogenic detection, Alexa Fluor 555-conjugated donkey anti-rabbit IgG antibody (ThermoFisher Scientific), Alexa Fluor 555-conjugated donkey anti-goat IgG antibody (Abcam), and/or FITC-conjugated donkey anti-Chicken IgY antibody (Abcam) for fluorescence detection. Counter-staining was performed with hematoxylin or 4′,6-diamidino-2-phenylindole (DAPI; ThermoFisher Scientific). The images of the sections were captured using a light microscopy (Nikon eclipse 80i) or a confocal laser scanning microscopy (Nikon eclipse Ti-E).

### Western Blotting

The intestinal crypts were homogenized and total protein was extracted using radioimmunoprecipitation assay (RIPA) buffer. Protein concentrations were determined using the bicinchoninic acid assay (BCA) method (ThermoFisher Scientific), normalized for all samples and separated on NuPAGE Novex 4–12% Bis-Tris gels (ThermoFisher Scientific). Subsequently, the proteins were transferred to polyvinylidene difluoride membranes (ThermoFisher Scientific), blocked with 5% skimmed milk in tris-buffered saline Tween (TBST), and incubated overnight with goat anti-C3/C3 fragments antibody (EMD Millipore) at a dilution 1:1000, rabbit anti-Ki67 antibody (Abcam) at a dilution 1:500, rabbit anti-Lgr5 antibody (ThermoFisher Scientific) at a dilution 1:1000, goat anti-C3aR1 antibody (Santa Cruz Biotechnology) at dilution a 1:500, and mouse anti-β-actin antibody (Sigma-Aldrich) at dilution a 1:5000 in TBST containing 5% skimmed milk. After three washes with TBST, the membranes were incubated with appropriate HRP-conjugated secondary antibodies (Santa Cruz Biotechnology). Protein bands were visualized using a chemiluminescent ECL™ detection kit (GE Healthcare).

### Statistical Analysis

Data are presented as means ± SEM. Statistical significance of differences was determined using Student’s *t*-test for comparison between two groups or by two-way analysis of variance with Berferroni correction for multiple comparisons. A *p*-value of <0.05 was considered statistically significant.

## Results

### C3a Promotes Intestinal Organoid Formation

**Figure 1:**
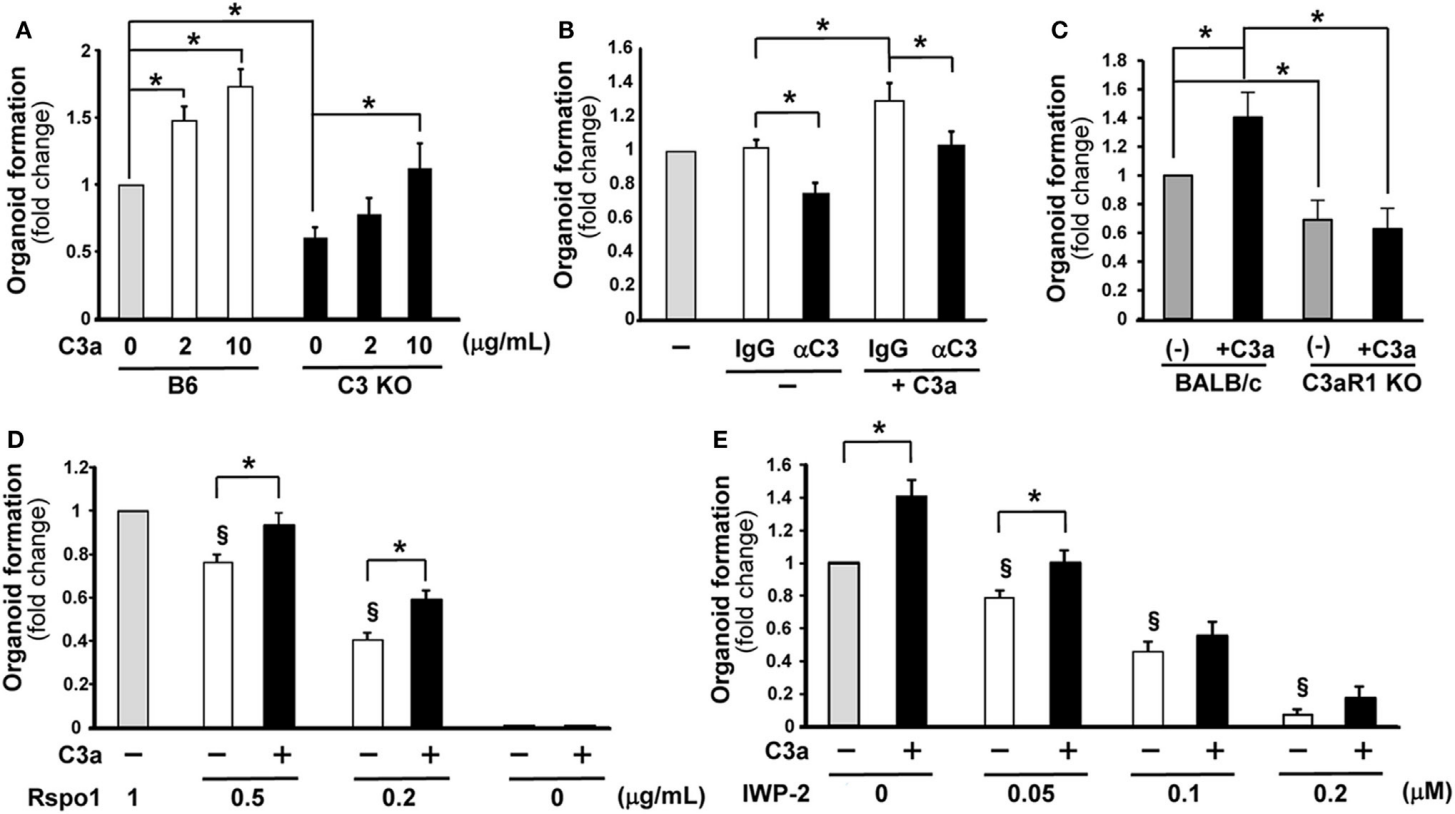
C3a promotes organoid formation. The number of organoids formed in wells 7 days were counted after the initiation of culture under various conditions. The values represent fold change that were calculated based on the number of organoids grown under control conditions. **(A)** Organoid formation was compared between C3-deficient (C3 KO) and B6 mice in the absence or presence of C3a in the medium at the concentration indicated below the horizontal axis (*n* = 7 mice in each group) *p < 0.05. **(B)** The effect of the presence of anti-C3a/C3 antibody (αC3) in the culture medium on organoid formation. Isotype control IgG or anti-C3a/C3 antibody was added in the cultures. *n* = 7 mice in each group, *p < 0.05. **(C)** The effect of C3a administration in the culture medium on organoid formation was evaluated using C3aR1-deficient mice and wild-type (BALB/c) mice. *n* = 5 mice in each group, *p < 0.05. **(D)** R-spondin 1 was added in the medium at the indicated concentrations with or without C3a and the effect on organoid formation was evaluated (*n* = 7 mice in each group). Rspo1, R-spondin 1. *p < 0.05 between the groups. ^§^*p* < 0.05 against 1 g/mL of R-spondin 1 without C3a. **(E)** The effect of the Wnt inhibitor IWP-2 on organoid formation was assessed in the absence or presence of C3a in the medium (*n* = 7 mice in each group). *p < 0.05 between the groups. ^§^*p* < 0.05 against the control conditions where neither C3a nor IWP-2 was present.

C3a is a key modulator of the inflammatory response to infectious and non-infectious insults (16) and promotes organ regeneration (17) yet it is unknown whether it is involved in intestinal stem cell function and organoid formation. To assess the involvement of C3a in intestinal regeneration, a “mini-gut” intestinal organoid assay was developed. The total number of organoids with multiple buds in each culture well was evaluated 7 days after the initiation of the culture using intestinal crypt cells from wild-type or C3-deficient mice. The number of organoids in cultures originated from C3-deficient mice was significantly lower compared to the number of organoids generated from the wild-type (B6) mice (Figure 1A), suggesting a role for C3 in the robust formation of intestinal organoids. Because intestinal cells produce C3 which may be cleaved by endogenous cathepsins B and L to produce C3a (14) we asked whether C3a can enhance organoid formation. We noted that C3a added to the culture medium significantly enhanced organoid formation in cultures of crypt cells from wild-type mice in a dose-dependent manner (Figure 1A). We observed a similarly statistically significant enhancing effect in organoid formation when we added C3a in cultures of crypt cells from C3-deficient mice (Figure 1A), indicating that extracellular C3a affects the growth of intestinal stem cells.

To further confirm the involvement of C3a in the growth of intestinal organoids, an anti-C3/C3a neutralizing antibody or its isotype control were added to the medium of wild-type mouse-derived organoid cultures. In the absence of added C3a, the anti-C3/C3a Ab (12, 13) significantly decreased organoid formation (Figure 1B). The enhancing effect of C3a in organoid formation was abolished in the presence of anti-C3/C3a antibody (Figure 1B). To demonstrate that the enhancing effect of C3a is canonical through the C3aR1, we set up organoid cultures using crypt cells from C3aR1-deficinet mice and their genetic background control BALB/c mice. Organoid formation from cells of C3aR1 KO mice was reduced when compared with that of BALB/c mice. Addition of C3a in culture media increased the organoid formation in BALB/c mice; however, we did not observe any changes in organoid formation in C3aR1 KO mice after the addition of C3a (Figure 1C).

Wnt signaling is one of the crucial pathways to maintain intestinal stem cell growth. The presence of the R-spondin 1, which stabilizes the Frizzled receptor for Wnt binding, in the organoid culture media is necessary for the generation of intestinal organoids (18). When the concentration of R-spondin 1 was limited to one-half of the standard concentration present in the organoid culture medium (0.5 µg/mL), the number of organoids was significantly decreased compared to that observed in the medium containing R-spondin 1 at the standard concentration of 1 µg/mL. Interestingly, the presence of C3a in the culture medium which contained one-half of the standard concentration of R-spondin 1 rescued the formation of organoids to normal levels (Figure 1D). The presence of extrinsic C3a increased significantly organoid formation even when the concentration of R-spondin 1 was lowered in the culture medium to 0.2 µg/mL (Figure 1D). When R-spondin 1 was completely excluded from the culture medium, the presence of C3a did not enable organoid formation (Figure 1D). These findings suggest that C3a supports the function of R-spondin 1. Next, we blocked Wnt signaling with IWP-2, a Wnt inhibitor. The presence of IWP-2 in the culture medium decreased the number of organoids formed in a dose-dependent manner. The addition of C3a enhanced organoid formation only when IWP-2 was added at a low concentration (0.05 µM) and the beneficial effect was readily abolished in the presence of IWP-2 at a higher concentration (0.1 µM) (Figure 1E). The Wnt signaling inhibition experiments provide further evidence that C3a supports Wnt signaling in the growth of intestinal stem cells.

### C3a Induces Ki67 and Lgr5 Expression in Intestinal Organoids

**Figure 2:**
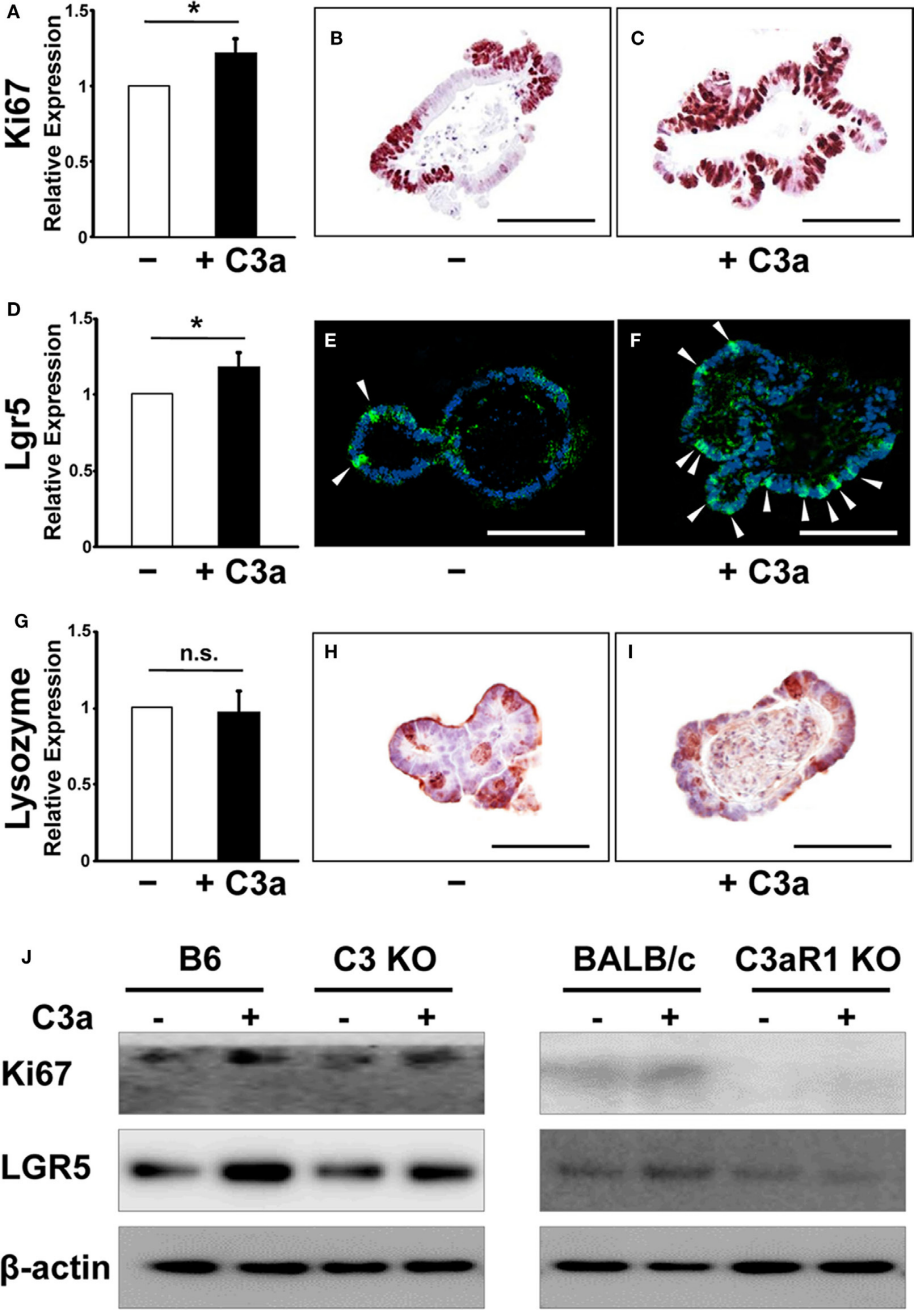
The expression of Ki67 and leucine-rich repeat G-protein-coupled receptor 5 (Lgr5) were enhanced in organoids in response to C3a. mRNA expression of Ki67 **(A)**, Lgr5 **(D)**, and Lysozyme **(G)** was compared in organoids in the absence or presence of C3a in the medium. *p < 0.05. n.s., not significant (*n* = 3 mice in each group). Immunohistochemistry was performed to detect expression of Ki67, Lgr5, and Lysozyme in organoids in the absence [**(B,E,H)**, respectively] or presence [**(C,F,I)**, respectively] of C3a. Arrows in E and F indicate Lgr5 intestinal stem cells. Scale bar = 100 µm (*n* = 3 mice in each group). Protein expression of Ki67 and Lgr5 **(J)** were compared in organoids from B6 mice, C3 KO mice, BALB/c mice, and C3aR1 KO mice by western blotting in the absence or presence of C3a in the medium. β-actin was used as a protein loading control (*n* = 3 mice in each group).

Next, we asked whether C3a alters the expression of proliferation (Ki67) and stemness (Lgr5) markers. C3-deficient organoids were formed in the presence or the absence of C3a (10 µg/mL) in the culture medium and total RNA was isolated 7 days later and analyzed by RT PCR. The expression of Ki67 mRNA was significantly upregulated in response to C3a administration in organoids (Figure 2A). Immunohistochemistry showed an increased number of Ki67-positive cycling cells in organoid culture in the presence of C3a (Figures 2B,C). Similarly, the presence of C3a in the culture medium significantly induced the expression of Lgr5 mRNA (Figure 2D). The population of Lgr5 positive cells increased impressively in the C3a-treated organoid cultures (Figure 2E vs. Figure 2F). By contrast, the presence of C3a did not affect the expression of lysozyme 1 mRNA (Figure 2G), a marker of Paneth cells that function to support the intestinal stem cell maintenance by supplying trophic factors such as Wnt (19). Immunostaining for lysozyme did not show any difference between organoids cultured in the presence or absence of C3a (Figures 2H,I). The addition of C3a significantly enhanced the expression of Ascl2 (Figure S1 in Supplementary Material), a transcription factor that is induced through Wnt stimulation and reinforces intestinal stem cell identity (20). To explore whether the effect of C3a in proliferation and stemness involves C3aR1, we studied the expression of Ki67 and Lgr5 in organoids generated from C3 KO mice and their corresponding B6 control mice, as well as in organoids from C3aR1 KO mice and their corresponding control BALB/c mice. Our finding of increased expression of both Lgr5 and Ki67 in the organoids from the control and C3 KO mice but not from the C3aR1 KO mice (Figure 2J) indicates that C3a promotes cell proliferation and stemness through C3aR1.

### Lgr5 Intestinal Stem Cells Express C3/C3a

**Figure 3:**
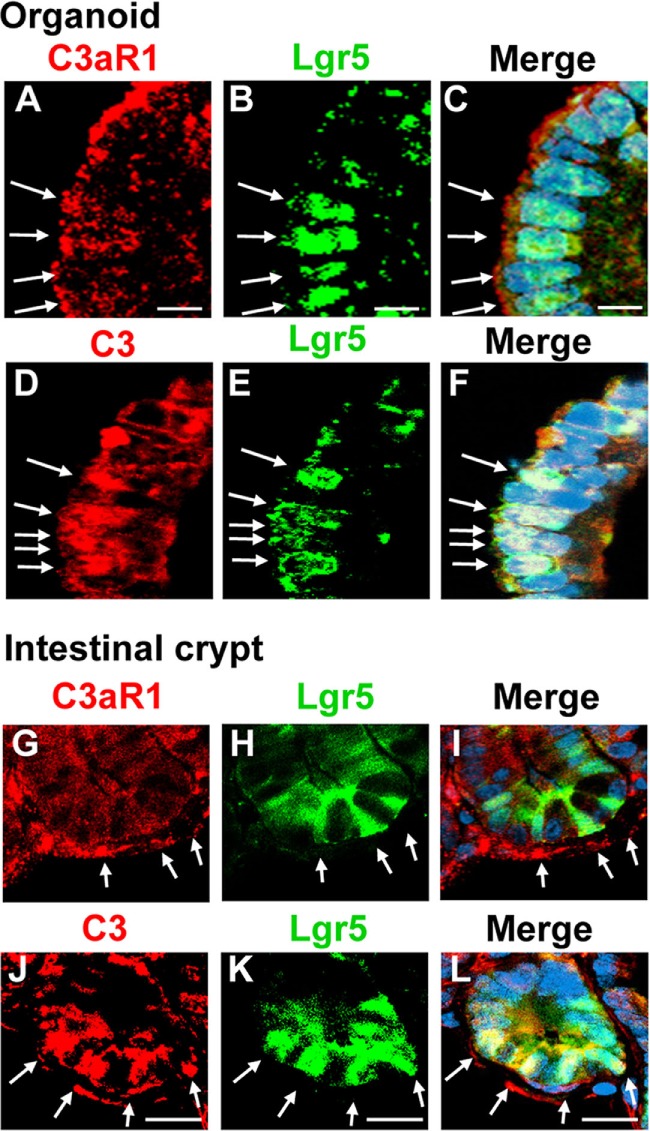
C3aR1 and C3 are expressed by Lgr5^+^ intestinal stem cells. Immunohistochemistry was performed to visualize the distribution of C3aR1 and C3 in *in vitro* organoids [**(A,D)**, respectively] and in *in vivo* intestinal crypts [**(G,J)**, respectively] using confocal microscopy. Lgr5^+^ intestinal stem cells were identified by detecting GFP **(B,E,H,K)** in *Lgr5.egfp.IRES.CreERT2* knock-in mice. The sections were counterstained with DAPI (images not shown). **(C,F,I,L)** are the merged images. Arrows indicate Lgr5 intestinal stem cells. Scale bar = 10 µm **(A–F)**, 20 µm **(G–L)** (*n* = 3 mice in each group).

To establish in a definitive manner the expression of C3 and C3aR1 by intestinal stem cells, we performed experiments using *Lgr5.egfp* mice. Tissue sections were prepared from intestinal organoids and small intestine of *Lgr5.egfp* mouse in which Lgr5^+^ intestinal stem cells can be identified through the detection of EGFP signal to analyze the distribution of C3 and C3aR1. C3aR1 was broadly expressed on the cell membrane of a variety of organoid cells, including Lgr5^+^ intestinal stem cells (Figures 3A–C). It was also distributed ubiquitously in the intestinal crypt that harbors Lgr5^+^ intestinal stem cells and Paneth cells (Figures 3G–I). C3 was detected in Lgr5^+^ intestinal stem cells in organoids (Figures 3D–F) as well as in intestinal crypts (Figures 3J–L), suggesting that intestinal stem cells can supply C3a to support the growth of intestinal stem cells in an autocrine manner.

### C3 and C3aR1 Are Induced in Intestinal Crypts in Response to Intestinal or Ischemia/Reperfusion

**Figure 4:**
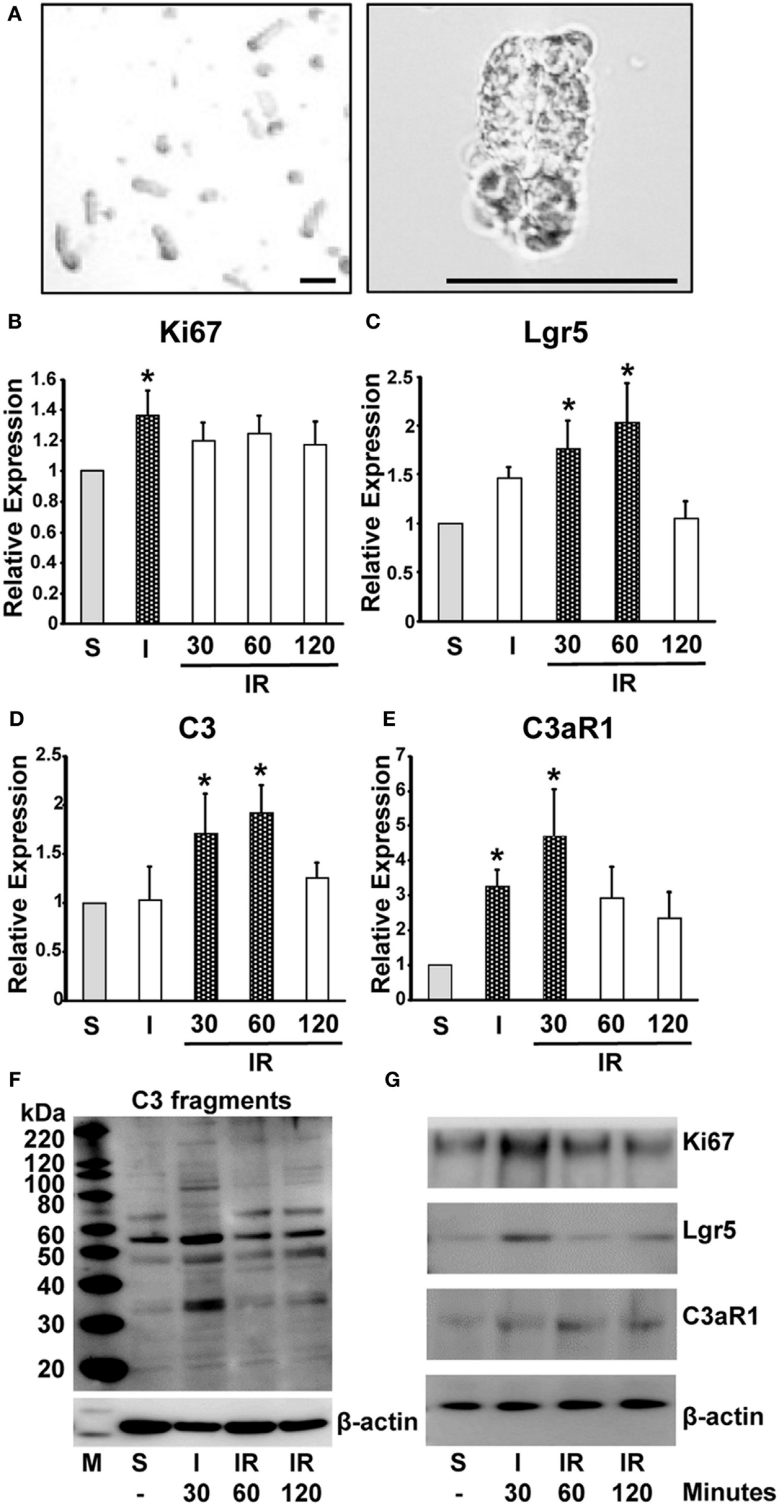
Induction of mRNA and protein expression of Ki67, Lgr5, C3 fragments, and C3aR1 in intestinal crypts during ischemia/reperfusion. Mice were subjected to 30 min ischemia followed by reperfusion. Intestinal crypts were collected from the dissected intestine **(A)** (left, low and right, high magnification. Length of bar = 100 µm). mRNA was isolated from the crypts to evaluate the expression of Ki67 **(B)**, Lgr5 **(C)**, C3 **(D)**, and C3aR1 **(E)** by real-time PCR. *n* = 5 mice in each group. Total protein was extracted from crypt cells to evaluate expression of C3 fragments **(F)**, Ki67, Lgr5, and C3aR1 **(G)**. β-actin was used as a protein loading control. *n* = 3 mice in each group. S, sham; I, ischemia only for 30 min; IR, ischemia for 30 min and reperfusion for the indicated time.

Our data have so far demonstrated that C3a has a supportive role in “mini-gut” formation *in vitro*. We sought to produce evidence that C3a is involved in intestinal regeneration following injury. Expression of C3 and C3aR1 mRNA was evaluated along with mRNA of Ki67 and Lgr5 in intestinal crypts (Figure 4A) obtained from mice subjected to intestinal ischemia (30 min) or ischemia followed by reperfusion (30, 60, or 120 min). Expression of Ki67 mRNA was increased significantly 30 min after the initiation of ischemia and continued for 2 h of reperfusion (Figure 4B). The expression of Lgr5 was significantly increased at 30 min and 1 h after reperfusion (Figure 4C). Interestingly, the timing of significant increase in C3 mRNA expression corresponded to that of Lgr5 (Figure 4D). C3aR1 expression was also increased significantly (peaked at 30 min of reperfusion) and prior to that of C3 mRNA (Figure 4E). Using Western blotting, we evaluated the protein levels of the above molecules in the intestinal crypts under sham and ischemia, or ischemia/reperfusion conditions. C3 fragments, Ki67, Lgr5, and C3aR1 increased primarily after 30 min of ischemia and remained relatively high during reperfusion (Figures 4F,G). We also examined Lgr5 expression under the same conditions in C3 KO and C3aR1 KO mice and found that they were increased in the KO mice similarly to the corresponding controls, suggesting that Lgr5^+^ cells can also be induced by ischemia in a C3/C3aR1-independent fashion (Figure S2 in Supplementary Material).

### C3aR1 Deficiency Does Not Mitigate Intestinal Tissue Damage after Ischemia and Reperfusion

**Figure 5:**
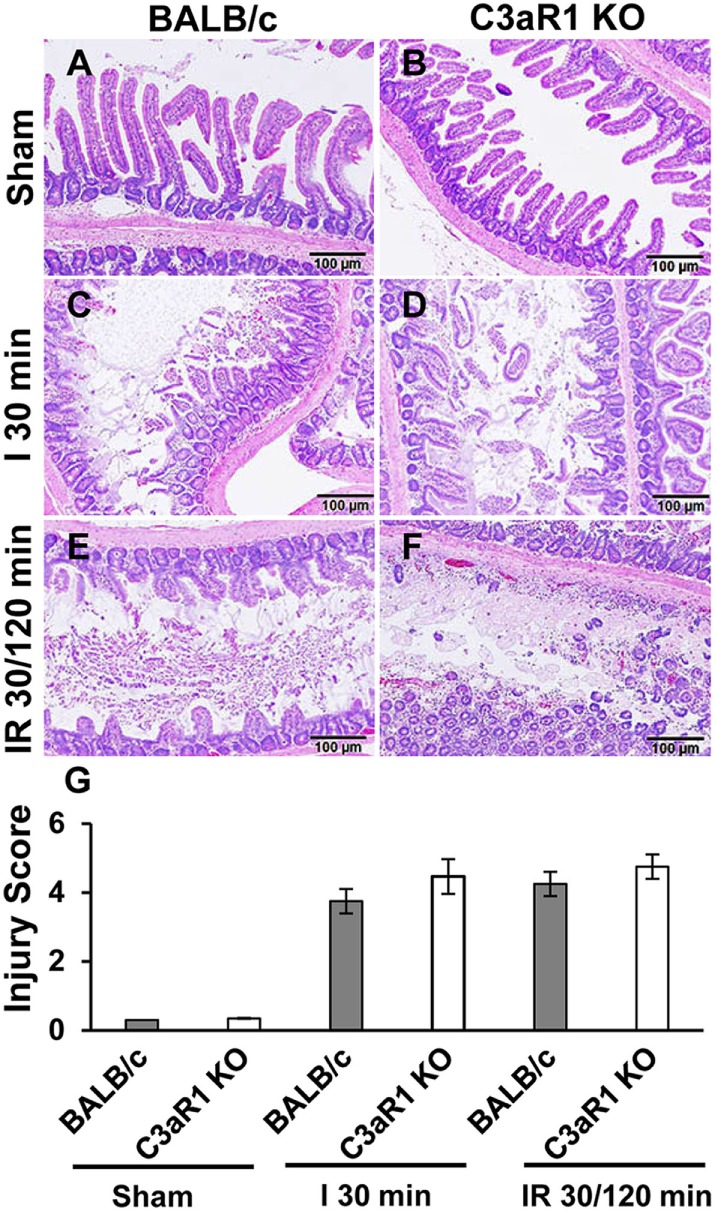
Mesenteric ischemia and ischemia followed by reperfusion enhances tissue damage in wild-type (BALB/c) mice and C3aR1-deficient mice (C3aR1 KO). H&E-stained sections of sham-operated small intestine from BALB/c mice **(A)** and C3aR1 KO mice **(B)**. Mice were subjected to superior mesenteric artery occlusion for 30 min (I 30 min) to induce ischemia in BALB/c mice **(C)**, and C3aR1 KO mice **(D)** followed by 120 min reperfusion (IR 30/120 min) in BALB/c mice **(E)**, and C3aR1 KO mice **(F)**. Scale bars, 100 µm. Cumulative data of injury scores derived from three independent experiments on separate days (three mice per group) are shown in **(G)**. Error bars represent SD.

Previously, we demonstrated a significant reduction in the intestinal injury in C3-deficient mice compared with C3-sufficient mice after intestinal ischemia and reperfusion (14). To determine whether C3aR1 is involved in intestinal damage and repair during ischemia and reperfusion, we evaluated the severity of villus damage after 30 min of ischemia and 120 min of reperfusion in C3aR1-deficient mice and compared them with C3aR1-sufficient mice (BALB/c). Villus damage was not observed in either BALB/c or C3aR1 KO mice subjected to sham procedures (Figures 5A,B). Villus damage was noted in BALB/c and C3aR1 KO mice subjected to 30 min of ischemia (Figures 5C,D) or 30 min of ischemia followed by 120 min of reperfusion (Figures 5E,F). Cumulative data of ischemia- and ischemia/reperfusion-induced intestinal injury are shown in Figure 5G.

## Discussion

Prompt intestinal epithelial regeneration is required after extensive loss of villous epithelium induced by intestinal injury because prolonged breakdown of the epithelial barrier will allow toxic nutrients and pathogens to enter the body indiscriminately. Tissue injury is accompanied by inflammation and it is reasonable to consider that inflammation-defined molecules drive the regeneration machinery to maintain intestinal homeostasis (21). This concept is supported by our results that demonstrate that C3a enhances mini-gut generation *in vitro* and C3 production is up-regulated in intestinal crypts as an immediate response to ischemia/reperfusion injury. We previously reported that intracellular C3a is produced by the epithelial cells lining the villi in response to intestinal ischemia and exacerbates intestinal epithelial loss (14).

In this study, we show that organoid formation from intestinal crypts of mice deficient in C3 was reduced. More importantly, organoid formation was reduced in mice deficient in C3aR1 and it did not increase even when the cultures were supplemented with C3a suggesting that C3a/C3aR1 signaling is important in stem cell proliferation. Organoid formation enhancement by C3a appeared to support Wnt signaling. Pharmaceutical inhibition of the Wnt signaling or the absence of R-spondin1 mitigated the enhancing effect of C3a.

When crypt cells from B6, C3 KO, and BALB/c mice were exposed to C3a *in vitro* they exhibited increased proliferative capacity, as demonstrated by increased expression of Ki67 and upregulated the stem cell indicator Lgr5. By contrast, Ki67 was not expressed and did not increase in the presence of C3a in crypt cells obtained from C3aR1 KO mice. This suggests that C3aR1 signaling is important for Lgr5 expression and crypt cell proliferation. Basak et al. (22) reported that Lgr5^High^Ki67^+^ stem cells are in continuous cell cycle and are efficient in forming organoids, while Lgr5^Low^Ki67^−^ are in a quiescent state having lost their stemness and clonogenic ability. Our finding that C3aR1 KO organoids express low levels of Lgr5 and no Ki67 in conjunction with our finding that C3a induces Lgr5 and Ki67 only in C3aR1-expressing organoids supports our hypothesis that C3a/C3aR1 signaling is important in stem cell cycling and villus regeneration.

Although C3-deficient mice develop limited ischemia and ischemia/reperfusion injury (14), we found that C3aR1-deficient mice develop ischemia and ischemia/reperfusion injury, as also noted by others (23). Therefore, it appears that the complement system has a bidirectional effect on intestinal damage and repair. C3 is required for the activation of complement system and the pursuant destruction of cells through the formation of C5b-9 membrane attack complexes. By contrast, C3a is needed for the successful function of stem cells, which is important for intestinal regeneration and the absence of C3aR1 alone cannot mitigate injury. Local intracellular production and activation of complement has been noted recently and implicated in the regulation of cell function and in injury (24). In this communication, we extend this concept by showing the intestinal stem cells produce C3 and express C3aR1.

Increased expression of C3, C3aR1, Lgr5, and Ki67 in our *in vivo* studies in which crypts were isolated from B6 mice that underwent ischemia or ischemia followed by reperfusion indicates that C3a/C3aR1 signaling may be important in stem cell proliferation and tissue regeneration *in vivo*. Increased expression of C3aR has also been reported in mice subjected to permanent cerebral ischemia (25) corroborating such an interpretation. On the other hand, upregulation of Lgr5 was similarly increased in crypts isolated from C3aR1 KO and C3 KO mice that underwent ischemia or ischemia followed by reperfusion suggesting that in addition to C3a, other factors may contribute to stem cell cycling necessary for villus repair (26). To conclude, our studies introduce an inflammatory molecule, C3a, as an important promoter of intestinal stem cell function and regeneration and a key contributor to the regenerative process.

## Ethics Statement

All mice were maintained in the pathogen-free, AAALAC (Association for Assessment and Accreditation of Laboratory Animal Care International) accredited animal facility at Beth Israel Deaconess Medical Center. All experimental protocols were performed in accordance with the National Institute of Health guidelines for the use of experimental animals and were approved by the Institutional Animal Care and Use Committee, Beth Israel Deaconess Medical Center, Boston.

## Author Contributions

NM and AS performed experiments, analyzed data and contributed in writing manuscript. MG, PL, and MGT directed animal experiments. JL contributed in data analysis. GT and MT designed the experiments, wrote manuscript, and supervised the work.

## Disclaimer

The opinions or assertions contained herein are the private views of the authors, and are not to be construed as official, or as reflecting the views of the United States Department of the Army or the Department of Defense.

## Conflict of Interest Statement

The authors declare that the research was conducted in the absence of any commercial or financial relationships that could be construed as a potential conflict of interest.
