# Supplementary material for: C3a Enhances the Formation of Intestinal Organoids through C3aR1
Source: Front Immunol. 2017 Sep 4;8:1046. doi: 10.3389/fimmu.2017.01046 (PMC5591398; doi:10.3389/fimmu.2017.01046)
Supplement: Supplementary file 1 [file image_1.pdf]

Supplementary Material

C3a enhances the formation of intestinal organoids through C3aR1

Naoya Matsumoto, Abhigyan Satyam, Mayya Geha, Peter H. Lapchak, Jurandir J. Dalle Lucca, Maria G. Tsokos, George C. Tsokos*

***Correspondence:** George C. Tsokos: gtsokos@bidmc.harvard.edu

Supplementary Figures

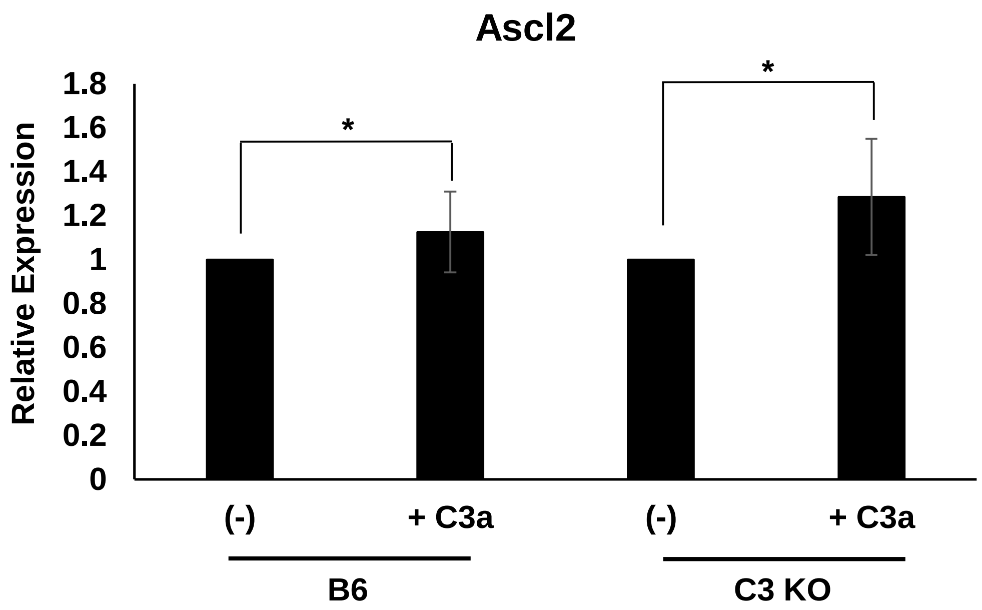

Supplementary Figure 1. Addition of C3a to organoid cultures significantly enhances mRNA expression of Ascl2. mRNA was isolated from organoids to evaluate the expression of Ascl2 by real-time PCR. n = 5 for B6 and n = 8 for C3 KO. mRNA expression of Ascl2 was compared in organoids in the absence or presence of C3a in the medium. * $p < 0.05$.

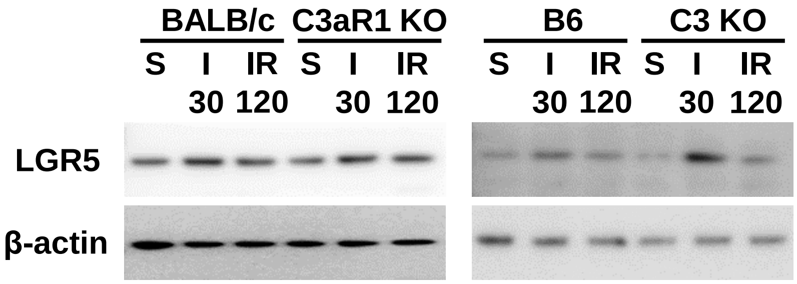

Supplementary Figure 2. Ischemia enhances Lgr5 expression. Mice (BALB/c, C3aR1 KO, B6 and C3 KO) were subjected to 30 min ischemia followed by 120 min of reperfusion. Intestinal crypts were collected from the dissected intestine and total protein was isolated to evaluate expression of Lgr5. β-actin was used as a protein loading control. n = 3 mice in each group. S: sham, I: ischemia only for 30 min, IR: ischemia for 30 min and reperfusion for 120 min.

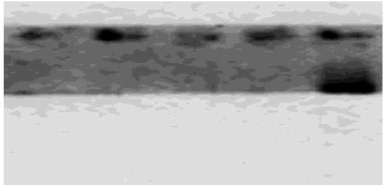

Ki67

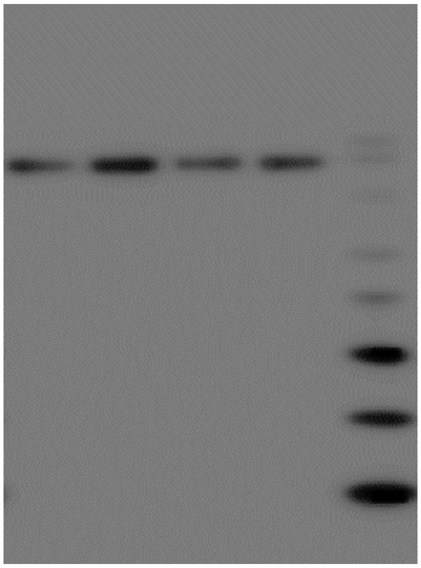

LGR5

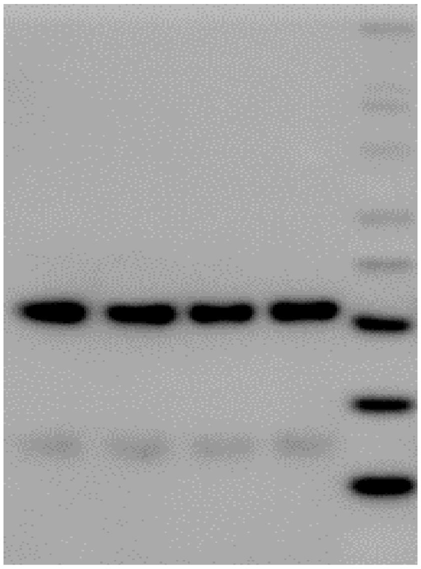

β-actin

Supplementary Figure 3. Ungrouped images for FIGURE 2 J (left panel). The Ki67 stained membrane was cropped before exposure for band development.

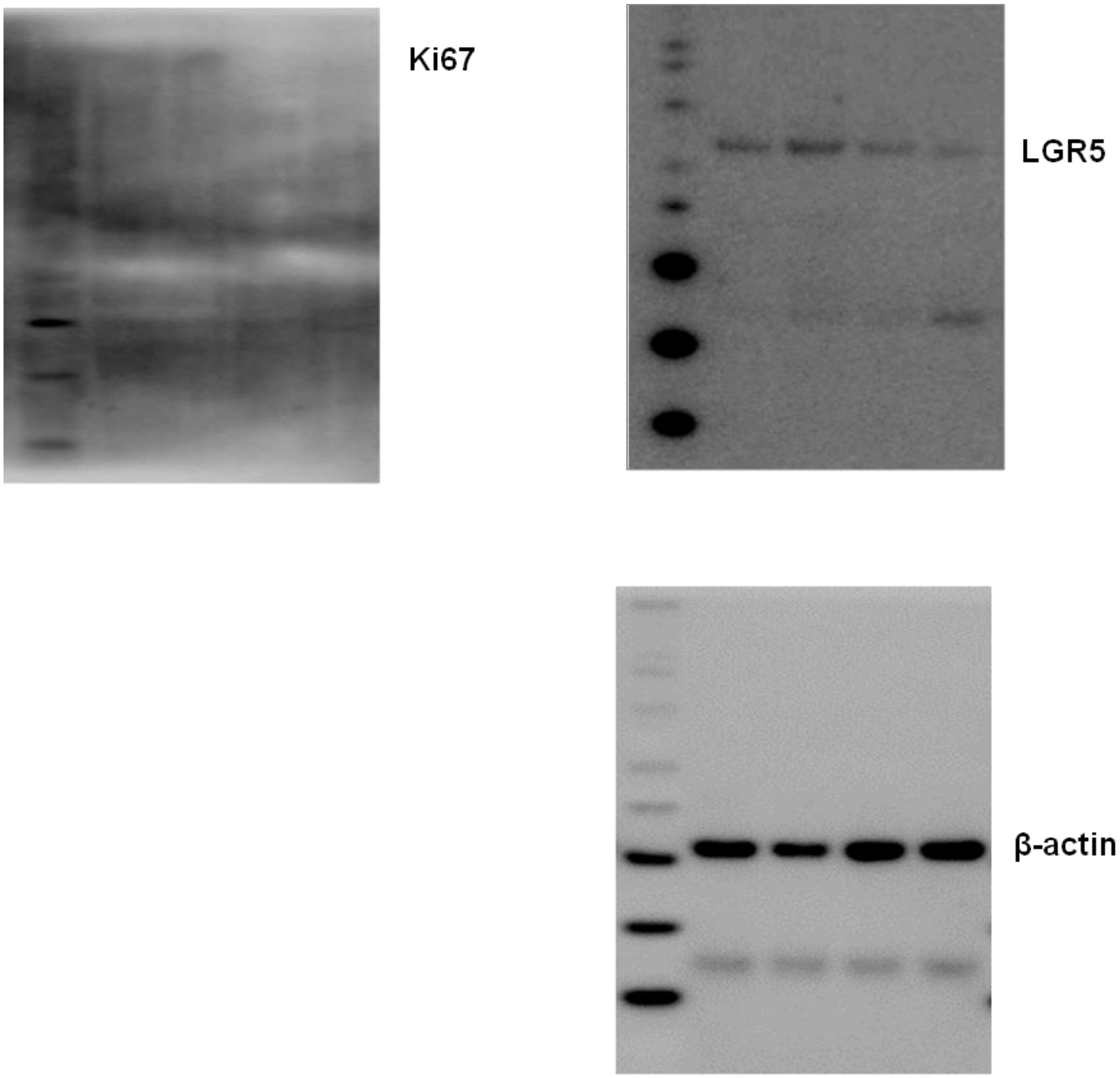

Supplementary Figure 4. Ungrouped images for FIGURE 2 J (right panel).

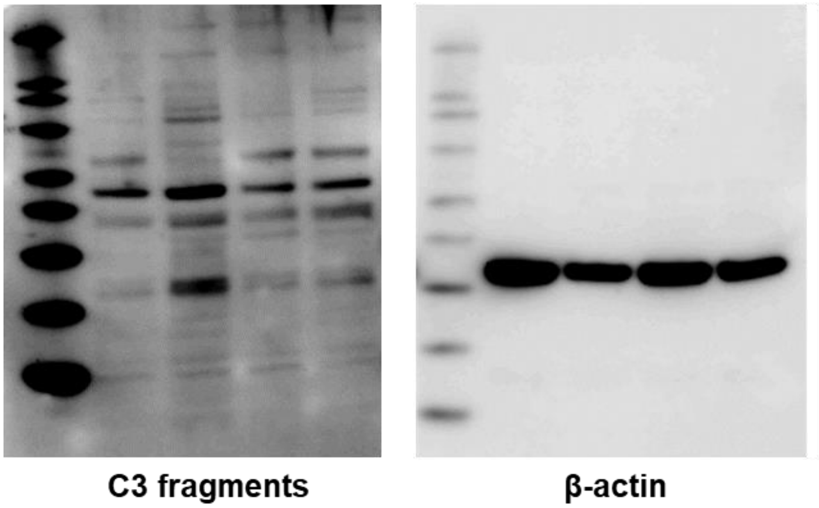

Supplementary Figure 5. Ungrouped images for FIGURE 4 F.

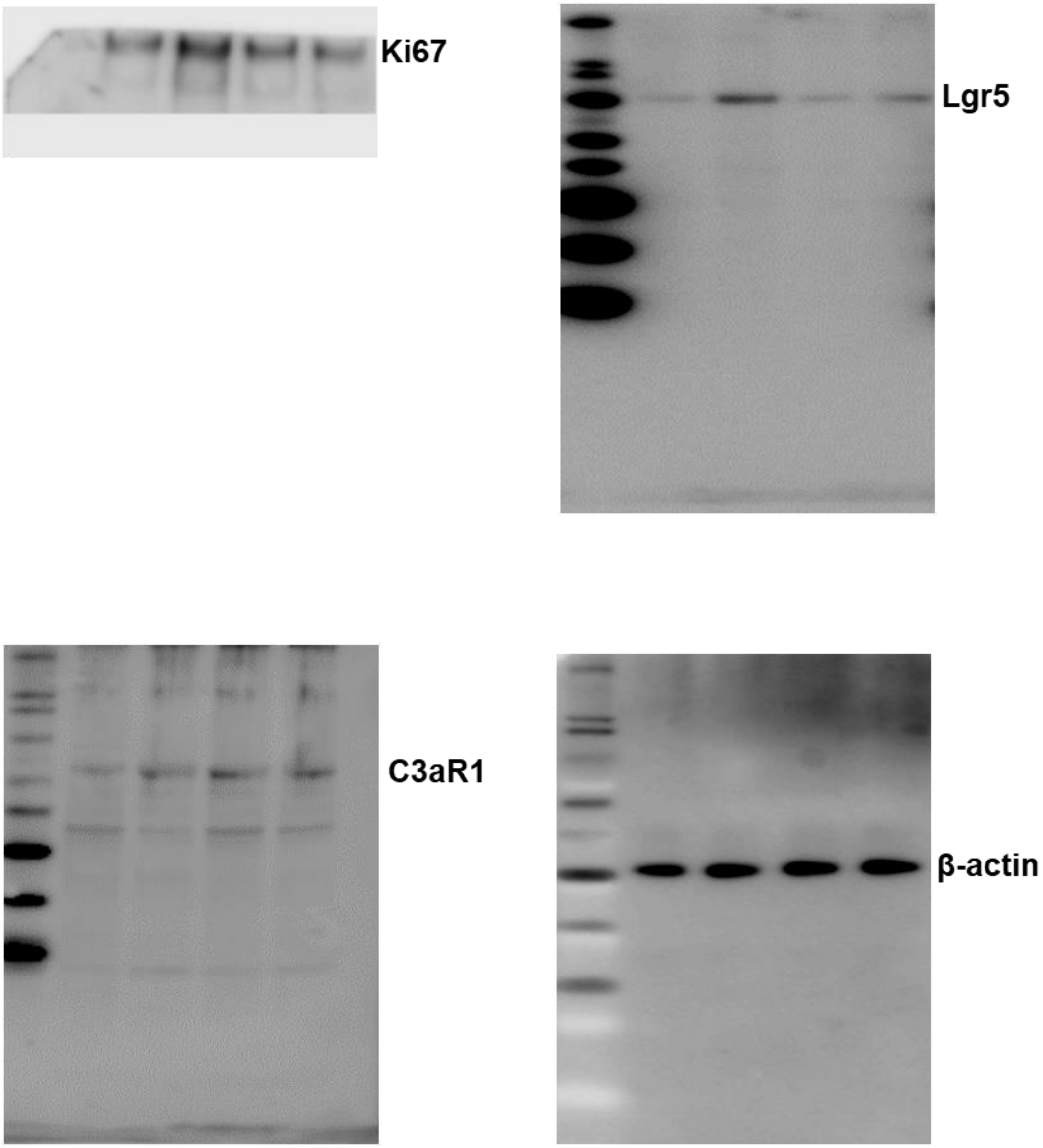

Supplementary Figure 6. Ungrouped images for FIGURE 4 G. The Ki67 stained membrane was cropped before exposure for band development.

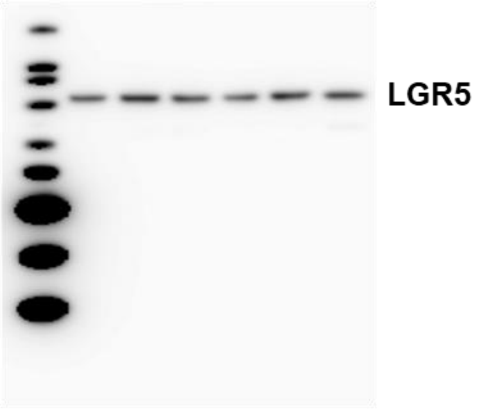

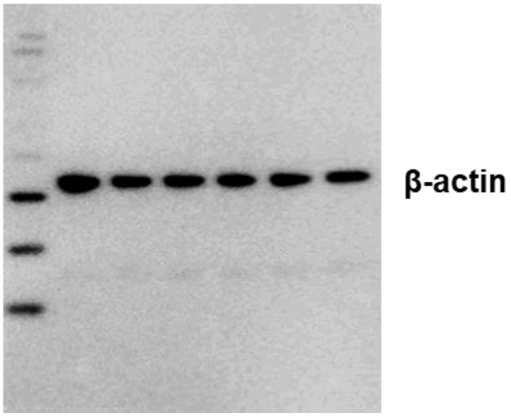

Supplementary Figure 7. Ungrouped images for Supplementary Figure 2 (left panel).

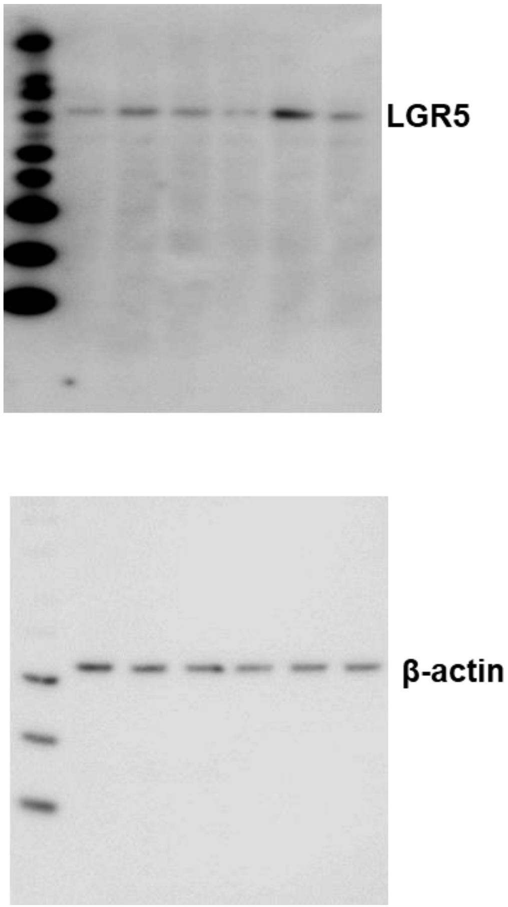

Supplementary Figure 8. Ungrouped images for Supplementary Figure 2 (right panel).
